# Cx43 channels and signaling via IP_3_/Ca^2+^, ATP, and ROS/NO propagate radiation-induced DNA damage to non-irradiated brain microvascular endothelial cells

**DOI:** 10.1038/s41419-020-2392-5

**Published:** 2020-03-18

**Authors:** Delphine Hoorelbeke, Elke Decrock, Maarten De Smet, Marijke De Bock, Benedicte Descamps, Valérie Van Haver, Tinneke Delvaeye, Dmitri V. Krysko, Christian Vanhove, Geert Bultynck, Luc Leybaert

**Affiliations:** 1 Physiology group, Department of Basic and Applied Medical Sciences, Ghent University, Ghent, Belgium; 2 Infinity Lab, IBiTech-MEDISIP, Department of Electronics and Information Systems, Ghent University, Ghent, Belgium; 3 Cell Death Investigation and Therapy Laboratory, Department of Human Structure and Repair, Ghent University, Ghent, Belgium; 4 Department of Physiology, Sechenov First Moscow State Medical University, Moskow, Russia; 5 Department of Cellular and Molecular Medicine, KU Leuven, Leuven, Belgium

**Keywords:** Cell signalling, Physiology

## Abstract

Radiotherapeutic treatment consists of targeted application of radiation beams to a tumor but exposure of surrounding healthy tissue is inevitable. In the brain, ionizing radiation induces breakdown of the blood–brain barrier by effects on brain microvascular endothelial cells. Damage from directly irradiated cells can be transferred to surrounding non-exposed bystander cells, known as the radiation-induced bystander effect. We investigated involvement of connexin channels and paracrine signaling in radiation-induced bystander DNA damage in brain microvascular endothelial cells exposed to focused X-rays. Irradiation caused DNA damage in the directly exposed area, which propagated over several millimeters in the bystander area. DNA damage was significantly reduced by the connexin channel-targeting peptide Gap26 and the Cx43 hemichannel blocker TAT-Gap19. ATP release, dye uptake, and patch clamp experiments showed that hemichannels opened within 5 min post irradiation in both irradiated and bystander areas. Bystander signaling involved cellular Ca^2+^ dynamics and IP_3_, ATP, ROS, and NO signaling, with Ca^2+^, IP_3_, and ROS as crucial propagators of DNA damage. We conclude that bystander effects are communicated by a concerted cascade involving connexin channels, and IP_3_/Ca^2+^, ATP, ROS, and NO as major contributors of regenerative signal expansion.

## Introduction

Ionizing radiation, in particular X-rays, is frequently used for diagnostic and therapeutic purposes. Although the precision of selectively irradiating tumor tissues is steadily increasing, radiation toxicity in healthy tissue remains a major issue affecting the potency of radiation treatment^1^. Tissue toxicity can be linked to vascular damage since microvascular endothelial cells, especially those forming the blood–brain barrier, are vulnerable to ionizing radiation. Microvascular endothelial cells respond with early production of reactive oxygen species (ROS) and DNA damage, including DNA double strand breaks (DSB), and late responses leading to apoptosis and senescence, resulting in ischemia, necrosis, and tissue fibrosis in normal surrounding tissues^2^. The damage inflicted to directly irradiated cells can be propagated to unexposed surrounding cells, known as the radiation-induced bystander effect, and mimics the direct effects of ionizing radiation encompassing induction of apoptosis, micronucleus formation, DSBs, and mutations^3,4^. Among the different effects of ionizing radiation exposure, DSBs are the most deleterious because of the high risk of misrepair^5,6^ and their potential to induce early and late radiation-induced effects^7^. A useful marker to detect DSBs is the Ser-139 phosphorylated form of the histone H2AX, γ-H2AX, which occurs rapidly after DSB formation^8,9^ and is followed by dephosphorylation (γ-H2AX disappearance) upon DSB repair. γ-H2AX foci not only appear in the irradiated area but also spread to bystander cells and form an elegant readout to investigate bystander signaling^10,11^. Although radiation-induced bystander effects have been investigated extensively, the signaling pathways, mechanisms, and molecules involved remain ill-defined. It also appears that considerable differences are observed depending on the cell type, radiation quality, and experimental approach used. Bystander effects are assumed to propagate via signals transmitted through gap junction channels, or via paracrine soluble factors released by irradiated cells^4,6^. Gap junctions are channels that directly connect two adjacent cells and are composed of two hemichannels, each formed by six connexin subunits. They allow the passage of substances up to ~1.5 kDa including atomic ions (Na^+^, K^+^, Ca^2+^) and important signaling or metabolic molecules like e.g., adenosine triphosphate (ATP), inositol-1,4,5-trisphosphate (IP_3_), glucose, and glutamate^12^. Before being incorporated into gap junctions, hemichannels are present as closed precursor channels that are known to open under ischemic or pro-inflammatory conditions^13^, oxidative stress^14^, decreased extracellular Ca^2+^, or increased intracellular Ca^2+ ^^15^. When open, hemichannels shunt the plasma membrane and facilitate bidirectional passage of below 1.5 kDa substances, leading to ionic shifts, depletion of important metabolites, or release of paracrine signaling molecules like ATP and glutamate^16^. Since connexins have a short half-life of several hours, they respond rapidly to stress and environmental changes. In humans 21 different connexin isotypes are known; vascular endothelial cells express Cx37, Cx40, and Cx43 depending on the vessel type^17^. The expression of Cx43, the most abundant connexin isotype in the human body^18^, increases in response to ionizing radiation, which results in enhanced gap junctional communication and contributes to radiation-induced bystander signaling in fibroblasts and epithelial cells^19,20^. Moreover, Cx43 hemichannels have been shown to be involved in ATP release in γ-ray-irradiated melanoma cells^21^ but the role of these channels in bystander signaling is currently unknown. Ca^2+^, ROS, and nitric oxide (NO) have been proposed as candidate bystander messengers, but ROS and NO are short-lived and have a limited effective diffusion distance making them unlikely as major propagating messengers of DNA damage^22–24^. Although the radiation-induced bystander effect gained significant interest over the past decade, the exact mechanisms underlying bystander propagation still remain incompletely understood especially with regard to the concerted action of the many signaling substances involved. Here, we aimed to elucidate the role of connexin channels and the Ca^2+^/ROS/NO signaling in brain endothelial cell bystander signaling. We hypothesized that ROS/NO may be responsible for the bystander effects, but the spreading is mediated by Ca^2+^ fluxes carried by extracellular ATP and intracellular IP_3_ facilitated by connexin channels.

## Materials and methods

### Cell cultures

The RBE4 (rat brain endothelial) cell line was kindly provided by Dr F. Roux (Neurotech, Evry, France). The RBE4 cells were grown on collagen (rat-tail collagen; Roche diagnostics, Vilvoorde, Belgium) coated recipients in alfa-MEM + F10 (1/1) medium supplemented with 0.6% geneticin, 1% L-glutamax, 10% fetal bovine serum (FBS, Gibco, Invitrogen, Merelbeke, Belgium), and 1 ng/mL human recombinant basic fibroblast growth factor (hbFGF, Roche diagnostics). Cells plated for the radiation experiments were grown without hbFGF. Next to this cell line, also primary brain microvascular endothelial cells (pBMECs) isolated from C57BL6 and C57BL/6 Cx43:^fl/fl^Tie2-Cre mice, were used. Primary BMECs were grown in DMEM (Gibco, Invitrogen, Merelbeke, Belgium) supplemented with 20% newborn calf serum (PAN Biotech, Aidenbach, Germany), 1% glutamax (Gibco, Invitrogen, Merelbeke, Belgium), 0.5% gentamicin (Gibco, Invitrogen, Merelbeke, Belgium), 1% vitamins, 2% amino acids, and 1 ng/mL hbFGF; hbFGF was removed from the cultures 24 h prior to irradiation. Primary BMECs cultures were grown on plates coated with matrigel (3.5 µg/cm²). Patch clamp experiments were performed on HeLa cells stably transfected with Cx43^25–27^, cultured in Dulbecco’s modified Eagle’s medium (Invitrogen, Ghent, Belgium), supplemented with 10% FBS, 2 mM glutamine, 10 µg/ml streptomycin, 10 U/ml penicillin, 0.25 µg/ml fungizone (Invitrogen, Ghent, Belgium), and 1 µg/ml puromycin (Sigma-Aldrich, Bornem, Belgium). HelaWT cells were grown in the medium without puromycin. Mouse Cx43 gene was cloned into the EcoRI/BamHI restricted cloning site of the expression vector pMJgreen. Cytomegalovirus promoter was used. The vector also contains a puromycin N-acetyltransferase gene encoding region.

### Agents

2ʹ,6ʹ-diamidino-2-phenylindole (DAPI), carbenoxolone (Cbx), N-acetyl-L-cystein (NALC), pyridoxalphosphate-6-azopehyl-2ʹ,4ʹ-disolfonic acid (PPADS), 2-(4-carboxyphenyl)-4,4,5,5-tetramethylimidazoline-1-oxyl-3-oxide potassium salt, 2-(4-carboxyphenyl)-4,5-dihydro-4,4,5,5-tetramethyl-1H-imidazol-1-yloxy-3-oxide (C-PTIO), and 4% formaldehyde were purchased from Sigma-Aldrich. 1.2-bis-(2-aminophynoxy)-ethane-N,N,Nʹ,Nʹ, tetraacetic acid acetoxy methyl ester (BAPTA-AM) originates from molecular probes (Invitrogen, Merelbeke, Belgium). Following connexin mimetic peptides were used: gap26 (VCYDKSFPISHVR, amino acids 64–76 from the first extracellular loop of Cx43) and TAT-gap19 (YGRKKRRQRRR-KQIEIKKFK, amino acids 128–136 in the second half of the CL of Cx43) obtained from Genosphere biotechnologies (Genosphere biotechnologies, Paris, France) with a purity >85%.

### Isolation of brain microvascular endothelial cells

Cortices from 10 to 12-week-old mice, were isolated by removing cerebellum, striatum, optic nerves, brain white matter, outer vessels, and meninges. After homogenization with a Dounce homogenizer in Washing Buffer B (WBB: Hank’s Balanced Salt Solution (HBSS), 10 mM HEPES (2-[4-(2-hydroxyethyl)piperazin-1-yl]ethanesulfonic acid), 0.35 g/L NaHCO_3_, 0.1% bovine serum albumin (BSA)), the homogenate was mixed with 30% dextran in WBB and centrifuged at 3000 × *g* for 25 min at 4 °C. The pellet containing the vascular component was then resuspended in WBB and filtered through a 60 µm NY60 Nylon Net Filter (Millipore, Darmstadt, Germany). Following centrifugation at 1000 × *g* for 7 min at room temperature, the pellet was digested in collagenase/dispase supplement with DNase I (Roche Diagnostics, Vilvoorde, Belgium) and Tosyl, lysin chloromethyl ketone (Sigma-Aldrich) for 33 min at 37 °C in a shaking water bath. The digested capillary suspension was then seeded after multiple washing steps on wells coated with matrigel or glass coated with Corning Cell-Tak (VWR, Leuven, Belgium).

### X-ray irradiation

A defined area of the cell dishes were exposed to X-rays (1 Gy or 20 Gy) by using a small animal radiation research platform (SARRP, Xstrahl, Camberley, UK, 220 kV, 13 mA), making use of a 3 × 3 mm collimator. Whole dish irradiation was done using a 10 × 10 cm broad-beam collimator, while focused irradiation was performed with a 3 × 3 mm collimator. A Gafchromic RTQA2 film, with a sensitivity of 0.02 Gy, was placed underneath the cell dishes in order to delineate the irradiated zone. Control experiments with cells exposed to 0.02 Gy did not produce any detectable effect on the γ-H2AX scores (data not shown), excluding the possibility that scattered irradiation not detected by the irradiated film would influence the results in the bystander area.

### γ-H2AX immunostaining and counting procedure

Cells were fixed for 25 min with 4% formaldehyde (VWR) and blocked for 30 min with blocking buffer (5% normal goat serum, 1% BSA), 0.2% Triton X-100 in PBS D+). Overnight incubation with primary antibody (1/500 anti-γ-H2AX in dilution buffer, 1/10 blocking buffer in PBS D+) was followed by a 1 h incubation with 1/400 XX-biotin-goat anti-mouse antibody in dilution buffer, followed by a 1 h incubation with 1/400 streptavidin-alexa 488 in PBS D+. Nuclei were stained with 1 µg/mL DAPI in PBS D+ for 10 min and cells were kept in PBS D+ supplemented with NaN_3_ at 4 °C. All steps except the overnight incubation that was carried out at 4 °C, were performed at room temperature, and cell dishes were rinsed thoroughly with PBS D+ between all incubation steps. Imaging was done with an automated BD Pathway 435 imaging system (×10 objective, 10 × 10 montage resulting in an overall image size of 8.5 × 6.5 mm). The border between the irradiated zone and the bystander area was defined as the full width at two thirds of maximal radiation.

We tested how counting γ-H2AX-positive nuclei over a large surface area was related to the more classical cell-based approach of quantifying the number of γ-H2AX foci per nucleus. To that purpose, we acquired high magnification (x63 objective) images and quantified the relative area occupied by γ-H2AX foci per nucleus in the irradiated zone. We found a linear relation between the low (×10 objective) and high magnification-based quantifications in the range of 0.1–1 Gy; at higher 10–20 Gy doses, the relation flattened (Supplementary Fig. 1). In the bystander zone, the fraction of γ-H2AX-positive nuclei was in the range of 4–12%, which fell within the linear range.

**Fig. 1:**
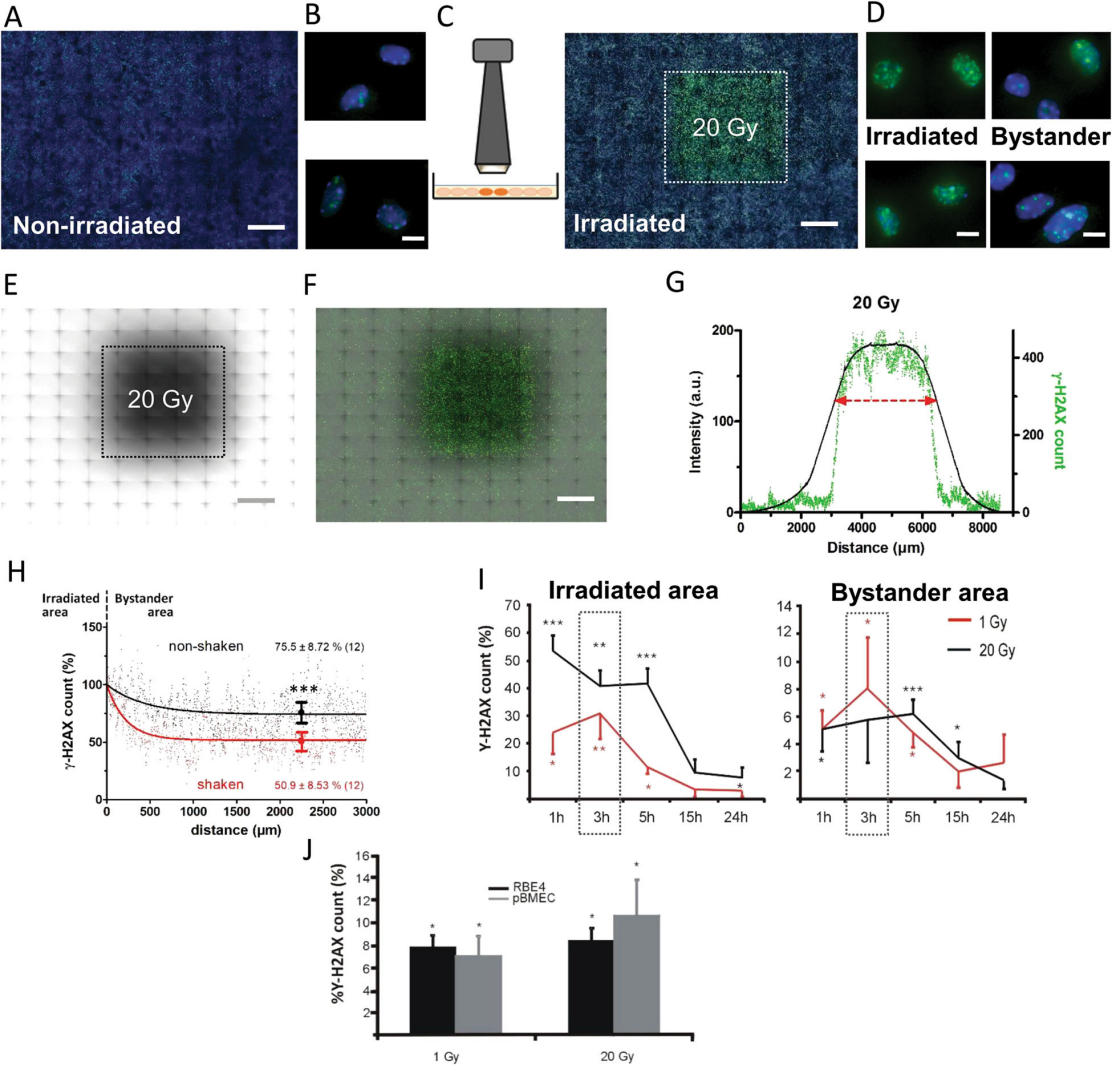
Investigating radiation-induced bystander effects in response to focused X-ray irradiation. **a** Control non-irradiated example image of RBE4 cells with DAPI nuclear staining in blue and background γ-H2AX staining in green (×10 objective; scale bar 1 mm). **b** Magnifications showing nuclear detail (×40; scale bar 10 µm). **c** Small aperture 3 × 3 mm collimator used for focused irradiation, with corresponding image taken 3 h post irradiation showing high density γ-H2AX foci (green) in the irradiated zone (dotted line; 20 Gy), and lower density in the surrounding bystander area (DAPI staining in blue; scale bar 1 mm). **d** Nuclear detail magnifications in irradiated and bystander areas (scale bar 10 µm). **e** A radiosensitive film placed underneath the cell dish was used to locate the irradiated zone (scale bar 1 mm). **f** Overlay of the radiosensitive film and the γ-H2AX image (scale bar 1 mm). **g** Demarcation of the irradiated zone was defined as the full width at two thirds of the maximum radiation intensity (red double arrowed line; ~3.4 mm wide) delineating the zone with the highest γ-H2AX foci count (raw counts, green curve). **h** Spatial profile of γ-H2AX foci in the bystander area in shaken and non-shaken cells 30 min after irradiation (raw counts normalized to 100% at the border with the irradiated zone). Gamma-H2AX counts averaged over the 1500–3000 µm interval were significantly different from each other. **i** Time dependence of γ-H2AX signal appearance in the irradiated and bystander areas (γ-H2AX counts relative to the number of nuclei and corrected for background in non-irradiated paired cell dishes) for 1 and 20 Gy irradiation. An asterisk (*) vs. non-irradiated (*n* = 5–11). **j** Gamma-H2AX counts in the bystander area recorded 3 h post irradiation were not different between RBE4 cells and pBMECs isolated from C57Bl6 mice. An asterisk (*) vs. non-irradiated.

**Fig. 2:**
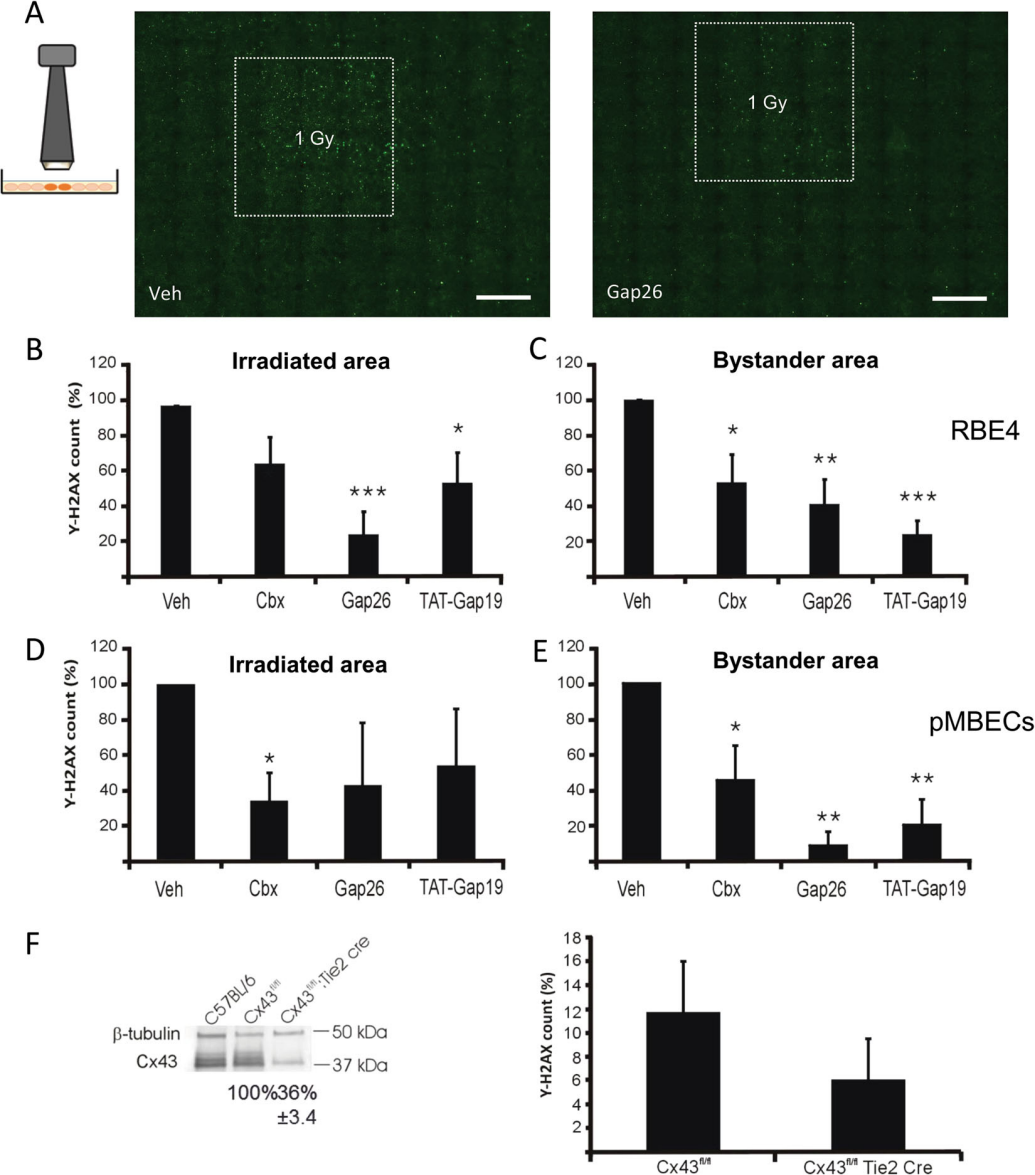
Connexin channel inhibitors reduce γ-H2AX responses in the irradiated and bystander areas. **a** Gamma-H2AX staining in RBE4 cells irradiated with the 3 × 3 mm collimator, treated with vehicle (left) or Gap26 (right; scale bars 1 mm). **b** Effect of connexin channel inhibition on γ-H2AX scores (normalized to vehicle) in RBE4 cells in the irradiated zone (*n* = 8–9). **c** Effect of connexin channel inhibition on bystander γ-H2AX (normalized to vehicle) in RBE4 (*n* = 8–9). **d**, **e** Experiments as in **b** and **c** but performed on pBMECs (*n* = 4–10). **f** γ-H2AX scores in the bystander area in pBMECs from C57BL/6 Cx43^fl/fl^:Tie2-Cre mice (*n* = 12); western blotting analysis (left) demonstrating decreased Cx43 expression in C57BL/6 Cx43^fl/fl^:Tie2-Cre.

We quantified the number of γ-H2AX foci-positive nuclei in the directly irradiated and bystander areas and expressed the count as a percentage relative to the number of nuclei and subtracted the percentage of background γ-H2AX signal in non-irradiated paired control cells for each experiment; this analysis was done with ImageJ. For counting γ-H2AX foci, a threshold was applied to remove background pixel noise below the foci level. Where applicable, results were normalized against vehicle (bar charts with a 100% vehicle bar without statistical variability or a horizontal line). Gamma-H2AX foci counts of Fig. 1g, h were calculated in a different way: for Fig. 1g, we calculated the summed raw counts of foci-positive vertical pixel columns for each pixel row position along the image *x*-axis. For Fig. 1h, counting was done as for Fig. 1g but data were normalized to the counts at the border of the irradiation zone (100%). The γ-H2AX foci images of Figs. 1a–d, f and 2a are raw unprocessed immunofluorescence images; foci images of Fig. 7e were processed by a digital dilation operation (ImageJ) to improve visibility of individual foci-positive dots.

### Gel electrophoresis and western blotting

RBE4 cells or pBMECs were seeded in 25 cm² falcons or 8 cm² petridishes. Lysates were made with RIPA (Cx43 and Cx37) and laemmli (Cx40) buffer. Protein concentration was determined using the BioRad DC protein assay kit (BioRad, Nazareth, Belgium). The lysate was separated by sodium dodecyl sulfate polyacrylamide gel electrophoresis (SDS-PAGE), over a mini-protean TGX stain-free gel (BioRad, Nazareth, Belgium) and transferred to a nitrocellulose membrane (Amersham, Buckinghamshire, UK). Membranes were blocked in TBS supplemented with 5% (Cx43 and Cx40) or 2% nonfat milk (Cx37) and 1% (Cx43 and Cx40) or 0.05% (Cx37) Tween20. The following primary antibodies were used: rabbit-anti-Cx43 (sigma), rabbit-anti-Cx37 (anti-rat and anti-mouse, Alpha Diagnositcs, Reinach, Switzerland), goat-anti-Cx40 (Santa Cruz), or rabbit-anti-β-tubulin antibody (Abcam, Cambridge, UK). Membranes were subsequently incubated with an alkaline phosphatase-conjugated goat anti-rabbit (Cx43, Cx37) or donkey anti-goat (Cx40) IgG antibody (Sigma-Aldrich). Detection was done using the nitro-blue-tetrazolium/5-bromo-4-chloro-3-indolyl-phosphate reagent (NBT/BCIP kit, Zymed, Invitrogen). Total protein staining was carried out with SYPRO Ruby protein blot dye (Invitrogen, Molecular Probes, Merelbeke, Belgium).

### Immunocytochemistry

For Cx immunoscytochemistry (Cx37, Cx40, and Cx37), fixation was as described for γ-H2AX immunostaining, followed by a 30 min blocking step with blocking buffer B (0.2% Tx100, 0.4% gelatin). Cells were incubated overnight with sheep anti-Cx37 (1/1000, Invitrogen), goat anti-Cx40 (1/50, Santa Cruz), or rabbit-anti-Cx43 (1/500), sigma) combined with rat anti-CD31 (combination of two antibodies, each 1/100, BD and Invitrogen) at 4 °C. In a next step, secondary antibodies were administered for 1 h (donkey anti-sheep alexa 594, chicken anti-goat alexa 594, goat anti-rabbit alexa 594, and goat anti-rat alexa 488, respectively, each 1/400 in blocking buffer B). Confocal images were taken with a Leica SP8 X confocal microscope (×63 water immersion objective) and analyzed using FiJi software.

### ATP measurements

For ATP measurements the supernatant present on the cells during irradiation was transferred to a 96-well plate for analysis. ATP was measured using a luciferin–luciferase assay kit following the manufacturer’s instructions (Sigma-Aldrich) in combination with a luminometer plate reader (Victor3 1420 multi label counter; Perkin Elmer, Zaventem, Belgium).

### Dye uptake

Cells were grown to near confluency in four-well plates and were incubated with 1 mM propidium iodide (PI) and 200 µM 10 kDa dextran-FITC before focal irradiation and dye uptake was measured 5 min post irradiation by measuring the fluorescence intensity of both dyes following image acquisition with a BD pathway 435 imaging system.

### Scrape loading and dye transfer (SLDT)

Confluent cell monolayers were washed three times with nominally calcium-free SLDT buffer (137 mM NaCl, 5.36 mM KCl, 0.81 mM MgCl_2_.6H_2_O, 5.55 mM D-glucose, 25 mM HEPES, pH 7.4). Cells were incubated for 1 min in SLDT buffer containing 400 μM 6-carboxyfluorescein; a linear scratch (one per dish) was made across the cell layer using a syringe needle, and the cells were left for another minute in the same solution. Cells were then washed six times with HBSS-HEPES and left for 15 min at room temperature, and images were taken with an inverted epifluorescence microscope equipped with a Nikon DS Ri1 camera.

### Electrophysiological recording

Subconfluent HeLa-Cx43 cell cultures were seeded on 13 mm diameter glass coverslips (Knittel Glaser, Novolab, Geraardsbergen, Belgium) and experiments were performed at subconfluency the next day. Recordings were performed in the presence of extracellular Ca^2+^ and Mg^2+^ and under conditions of K^+^-channel blockade with Cs^+^, Ba^2+^, and TEA^+^. Cells were bathed in a recording chamber filled with a modified Krebs–Ringer solution, consisting of (in mM): 150 NaCl, 6 CsCl, 2 MgCl_2_, 2 CaCl_2_, 5 glucose, 5 HEPES, 1 BaCl_2_, and 2 pyruvate, with pH adjusted to 7.4. The whole-cell recording pipette solution was composed of (in mM): 130 CsCl, 10 NaAsp, 0.26 CaCl_2_, 5 HEPES, 2 EGTA, 5 TEA-Cl, and 1 MgCl_2_, with an adjusted pH of 7.2. Free intracellular Ca^2+^ was 50 nM, as calculated with Webmax Standard (http://www.stanford.edu/~cpatton/webmaxcS.htm) software application. An EPC 7 PLUS patch clamp amplifier (HEKA Elektronik, Lambrecht/ Pfalz, Germany) was used to perform single-channel recordings. Data were acquired at 6 kHz using a NI USB-6221 data acquisition device from National Instruments (Austin, TX, USA) and WinWCP acquisition software (designed by Dr J. Dempster; University of Strathclyde, UK). All currents in whole-cell configuration were filtered at 1 kHz (seven-pole Bessel filter). For single-channel analysis, holding currents were subtracted from the recorded current traces, giving traces that only contained unitary current events. Unitary conductances were calculated from the elementary current transitions Δ*i* as: *γ* = Δ*i*/*V*_m_. From these data, we constructed all-point conductance histograms that displayed one or more Gaussian distributions. These were fit by a probability density function assuming independent channel opening^25,27–29^. Channel activity was quantified from the charge transfer *Q*_m_ associated with unitary currents; this was done by integrating the unitary current traces (i.e., a function of time) over the duration of the voltage step as: $Q_{\text{m}}=\int{i}{\text{d}}t$.

### Calcium imaging

Cells were seeded on glass coverslips (18 mm ø) coated with 3.5 µg/cm² Corning cell-tak (VWR, Leuven, Belgium) and ester loaded for 45 min with 10 µM Fluo-3-AM in HBSS-HEPES supplemented with 1 mM of probenecid and 0.01% of pluronic F127 at room temperature followed by de-esterification over 15 min. Imaging was performed using an inverted fluorescence microscope equipped with a ×40 oil immersion objective and an EM-CCD camera (QuantEM™ 512SC CCD camera, Photometrics, Tucson, AZ). For direct Ca^2+^ measurements the loaded cells were irradiated and transferred to the microscopy stage. For medium transfer, the loaded cells were superfused for 1 min with HBSS-HEPES (1 mM), followed by 4 min with HBSS-HEPES solution retrieved from irradiated cells. Fluorescence intensity changes in the cells were analyzed with custom-developed FluoFrames software (L. Leybaert, Ghent, Belgium). Ca^2+^ changes were quantified as the area under the curve (AUC) of the Ca^2+^ traces.

### ROS measurements

Cells were seeded in 96-well plates coated with collagen and broad-beam irradiated at different doses. For ROS measurements the cells were loaded with 10 µM of CM-H2DCFDA in HBSS-HEPES. Imaging was performed with a BD Pathway 435 microscope equipped with an automated imaging focus system, avoiding ROS generation associated with long exposures to excitation^30^. Results were corrected for the ROS produced in the irradiated medium alone.

### Cytotox Glo assay

To evaluate cell death, RBE4 cells were seeded in 96-well plates coated with collagen and irradiated using broad-beam at different doses. The CytoTox-Glo^TM^ Cytotoxicity assay (Promega, Madison, USA) was used following the manufacturers protocol. The CytoTox-Glo™ Cytotoxicity Assay uses a luminogenic peptide substrate, the AAF-Glo™ substrate, to measure dead-cell protease activity, which is released from cells that have lost membrane integrity. Measurements were performed with a luminometer plate reader.

### Electroporation

Cells were grown to near confluency on four-well plates coated with collagen. Cell monolayer dishes were washed three times with HBSS-HEPES 25 mM (pH 7.2–7.4) and subsequently three times with a low conductivity electroporation buffer (4.02 mM KH2PO4, 10.8 mM K2HPO4, 1.0 mM MgCl2, 300 mM sorbitol, 2.0 mM HEPES, pH 7.4). They were placed 400 μm underneath a two-wire Pt–Ir electrode on the microscopic stage and electroporated in the presence of a tiny amount of electroporation solution (10 μl) containing 100 µg/mL superoxide dismutase (SOD), 60 µM BAPTA, or 20 µM BH4-Bcl2 combined with 100 μM 10 kDa dextran Texas red (DTR). Control cell dishes were electroporated with solution containing only 100 μM DTR vehicle solution. Electroporation was carried out with 50 kHz bipolar pulses applied as trains of ten pulses of 2 ms duration each and repeated 15 times. The field strength was 100 V peak-to-peak applied over a 500 μm electrode separation distance. After electroporation, cells were thoroughly washed with HBSS-HEPES 25 mM followed by addition of CO_2_-independent medium (Invitrogen), which was also present on the cells during and following irradiation. The irradiated area was chosen away from the electroporation area (1555 ± 1468 µm border-to-border distance; *n* = 15) in order to investigate the effect of the blockers in the bystander area.

### Data and statistical analysis

Data are expressed as mean ± SEM, with “*n*” denoting the number of independent experiments. Multiple groups were compared by one-way ANOVA and a Bonferroni posttest, using GraphPad Instat Software (Graphpad Software). *P* < 0.05 was considered statistically significant. Statistical significance is indicated in the graphs by one symbol for *P* < 0.05, two symbols for *P* < 0.01, and three symbols for *P* < 0.001.

## Results

### X-ray-induced DNA damage is propagated from irradiated to non-irradiated bystander cells

To investigate the role of connexin-mediated intercellular communication in radiation-induced bystander responses, we irradiated a defined area of adherent brain microvascular endothelial cells. Both RBE4 cells, a rat brain microvascular endothelial cell line, as well as pBMECs isolated from mouse brains, grown to confluency, were used to that purpose. These cells express Cx37, Cx40, and Cx43 based on western blotting and immunocytochemical studies (Supplementary Fig. 2). Endothelial cell monolayers were locally exposed to X-rays (1 and 20 Gy) using a 3 × 3 mm collimator (Fig. 1), resulting in an irradiated area of ~9 mm^2^ within a total 190 mm² cell dish surface area. The irradiated zone was identified by a radiosensitive film positioned below the cell dish (Fig. 1e). After irradiation, γ-H2AX immunostainings were performed and imaged in a 55.25 mm^2^ large area that included the irradiated and surrounding non-irradiated zones. Overlays between the radiosensitive film and γ-H2AX staining allowed to distinguish irradiated and non-irradiated surrounding zones (Fig. 1f). As inferred from Fig. 1f, the irradiated but also non-irradiated zone showed clearly discernable γ-H2AX foci, which were further spatially quantified in terms of radiation intensity and γ-H2AX signal (Fig. 1g).The radiation intensity curve had smoothly varying slopes, while the γ-H2AX profile showed sharp falling edges at the collimator borders, indicating a threshold-like γ-H2AX response. The borders of the irradiated zone were defined based on the full width at two thirds of the maximal radiation intensity (red arrowed line in Fig. 1g), reliably delineating a zone of ~3.4 × 3.4 mm (11.6 mm²). After irradiation, cell dishes were transferred to the lab for subsequent analysis and we thus verified whether leaving the cells immobile would give different γ-H2AX scores in the bystander zone. We found that movement and associated cell dish shaking gave significantly lower γ-H2AX scores in the bystander zone (Fig. 1h) compared with the non-shaken condition (30 min after irradiation). This suggests involvement of paracrine bystander communication, with shaking disturbing an unstirred layer thereby diluting locally released bystander signaling molecules.

In a next experiment, we determined the kinetics of the γ-H2AX signal by counting γ-H2AX-positive nuclei expressing them relative to the total number of nuclei and background correcting them by subtracting relative counts from non-irradiated paired control cell dishes (γ-H2AX count expressed as a percentage). We observed a dose-dependent increase in γ-H2AX scores in the irradiated zone at 1–3 h after irradiation (1 Gy dose), followed by a gradual recovery over the following 24 h (Fig. 1i). We also observed increased γ-H2AX scores in the bystander area, which peaked at 3–5 h post irradiation followed by recovery 24 h later. Interestingly, γ-H2AX responses in the bystander zone were not different between 1 and 20 Gy irradiation (8 ± 3.8% vs. 6.7 ± 2.9%, respectively, 3 h post irradiation; *n* = 5–10) suggesting that bystander responses saturate between 1 and 20 Gy, as reported by others^31–33^. The γ-H2AX scores recorded 3 h post irradiation in the bystander area were not different in pBMECs compared with RBE4 cells (Fig. 1j). As 1 and 20 Gy gave equipotent bystander effects, we chose the lower 1 Gy dose for further studies.

### Gap junction and hemichannel inhibitors reduce bystander γ-H2AX scores

Paracrine factors and direct cell–cell communication via gap junctions are both involved in mediating bystander effects^4^. As such, not only gap junctions, but also hemichannels could contribute to the DNA damage propagation process. To investigate the contribution of both channel types, we applied several connexin channel inhibitors: the general connexin channel blocker Cbx, Gap26 that targets Cx43, Cx40, and Cx37^34^ and inhibits hemichannels within minutes and gap junctions with longer exposures^27^, and TAT-Gap19 that blocks Cx43 hemichannels within 2 min, without inhibiting gap junctions^25^ (reviewed in Leybaert et al.^35,36^). Cbx (50 µM), applied 30 min before 1 Gy irradiation and also present in the medium thereafter (30 min + 3 h post irradiation), significantly inhibited γ-H2AX-positive cell counts in the bystander zone while its effect in the irradiated zone were non-significant (RBE4 cells; Fig. 2b, c). By contrast, Gap26 and TAT-Gap19 (161 µM and 200 µM resp.; 30 min + 3 h post irradiation) significantly inhibited γ-H2AX counts in both irradiated and bystander zones. Control experiments with addition of the TAT translocation sequence lacking the active Gap19 moiety did not significantly affect the γ-H2AX counts in both zones (irradiated zone: 110 ± 24%; bystander zone: 104 ± 48%; *n* = 15). Experiments on primary isolated BMECs demonstrated significantly decreased γ-H2AX counts in both irradiated and bystander zones with Cbx, whereas Gap26 and TAT-Gap19 significantly inhibited only in the bystander zone (Fig. 2d, e). Both Cbx and Gap26 inhibit channels formed by different connexin isotypes, while TAT-Gap19 specifically targets Cx43-based hemichannels. To further substantiate the contribution of Cx43, we isolated pBMECs from C57BL/6 Cx43:^fl/fl^Tie2-Cre mice that have targeted Cx43 knockout in endothelial and hematopoetic cells under control of the Tie2 promoter. pBMECs derived from the latter animals displayed strongly decreased Cx43 expression compared with Cx43^fl/fl^ mice, but the bystander response 3 h post irradiation only showed a non-significant trend to decrease (Fig. 2f). Of note, western blotting analysis demonstrated a trend for increased Cx37 expression in pBMECs from C57BL/6 Cx43:^fl/fl^Tie2-Cre mice (Supplementary Fig. 3b), suggesting compensatory Cx37 upregulation which may mask the Cx43 knockout effect.

### Irradiation induces Cx43 hemichannel opening

**Fig. 3:**
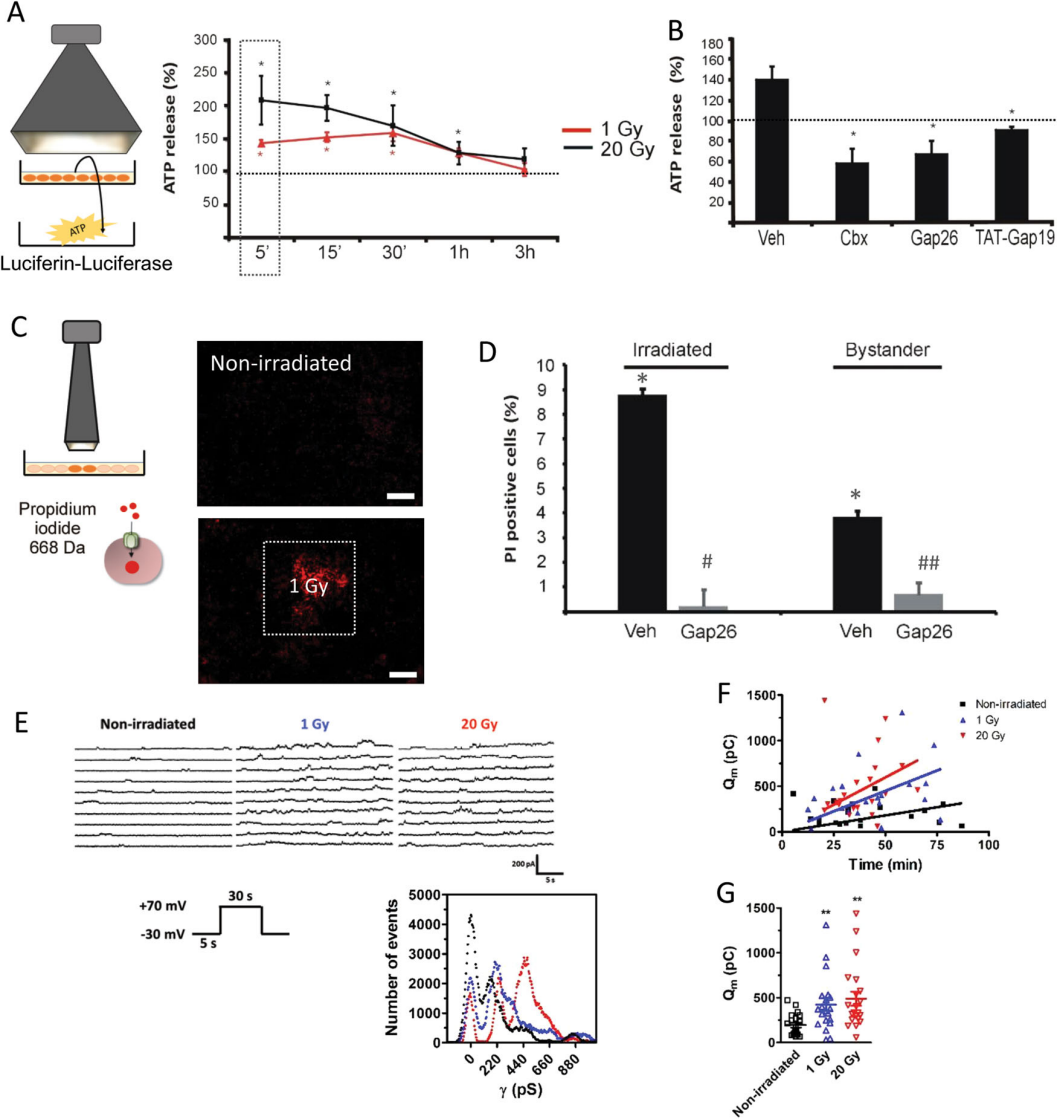
Irradiation triggers hemichannel opening. **a** Broad-beam irradiation triggers ATP release in RBE4 cells. The luciferin–luciferase signal is normalized to control non-irradiated (100% dotted line). An asterisk (*) vs. nonirradiated control (*n* = 6). **b** Summary data from the 5 min point 1 Gy irradiation (boxed area in **a**) illustrating the effect of connexin channel inhibition. An asterisk (*) vs. vehicle (*n* = 4). **c** Irradiation triggers propidium iodide dye uptake in the irradiated and bystander areas. Dye uptake 5 min post irradiation with the 3 × 3 mm collimator (1 Gy, scale bar 1 mm). **d** Summary data demonstrating radiation-induced dye uptake (relative to number of nuclei and background corrected for signal in non-irradiated cells) that is inhibited by Gap26 in both irradiated and bystander areas. An asterisk (*) vs. non-irradiated, The symbol “^#^” vs. vehicle, (*n* = 4). **e** Patch clamp experiments on HeLa-Cx43 cells demonstrating traces and matching all-point histograms depicting typical *V*_*m*_-induced (+70 mV, 30 s) Cx43 hemichannel unitary currents without irradiation and after 1 or 20 Gy irradiation. The histogram illustrates 220 pS and 440 pS peaks that are typical for Cx43 hemichannel opening. **f** Time course of unitary current activities for the conditions explained in **e**. Points represent membrane charge transfer (*Q*_*m*_) recorded at different time points after irradiation. Linear regression analysis demonstrates that the slopes increase from 0 to 20 Gy, indicating that hemichannel opening increases with time after radiation exposure. **g** *Q*_*m*_ summary data for repeated *V*_*m*_ steps to +70 mV (*n* = 5). An asterisk (*) vs. non-irradiated.

We examined whether X-rays open hemichannels by three different approaches: (i) measuring ATP release in the supernatant, (ii) determining the cellular uptake of the hemichannel-permeable dye PI, and (iii) patch clamp experiments. For ATP release, we irradiated RBE4 cells with a whole-field broad-beam collimator and measured ATP 5 min post irradiation. Both 1 and 20 Gy irradiation significantly increased extracellular ATP release compared with non-irradiated cell dishes and then gradually returned to baseline levels within 3 h (Fig. 3a). ATP release was inhibited by Cbx, Gap26, and TAT-Gap19 (added 30 min before and present until the end; Fig. 3b). In line with this, we found significantly increased cellular uptake of the hemichannel-permeable fluorescent dye PI in irradiated RBE4 cells, in both the irradiated and bystander zones 5 min post irradiation (1 Gy; Fig. 3c). PI uptake was significantly reduced by Gap26 in both areas (Fig. 3d). Irradiation did not affect the uptake of the hemichannel-impermeable dye 10 kDa dextran-FITC (data not shown), indicating membrane integrity was preserved; this was confirmed by the absence of detectable release of a high molecular weight protease (Supplementary Fig. 3e). For patch clamp experiments, both HeLa cells stably transfected with Cx43 (HeLa-Cx43) and HeLaWT were exposed to broad-beam irradiation (1 and 20 Gy) followed by rapid transfer to the electrophysiology setup for single-channel recording. Both 1 and 20 Gy doses resulted in increased unitary current events triggered by voltage steps from −30 to +70 mV applied to activate hemichannel opening (Fig. 3e). All-point histograms demonstrated a unitary conductance of ~220 pS typical for Cx43 hemichannels^25,27,37^. Potentiation of unitary current activities increased with post-irradiation time and radiation dose (Fig. 3f), and averaging the activity over all time points (range 5–87 min) demonstrated significantly increased membrane charge transfer (*Q*_*m*_) compared with non-irradiated controls (Fig. 3g). Of note, SLDT experiments did not show significant alterations of gap junctional coupling after 1 Gy irradiation (Supplementary Fig. 3c).

### Irradiation-induced intracellular Ca^2+^ elevation and ROS production lead to hemichannel opening

**Fig. 4:**
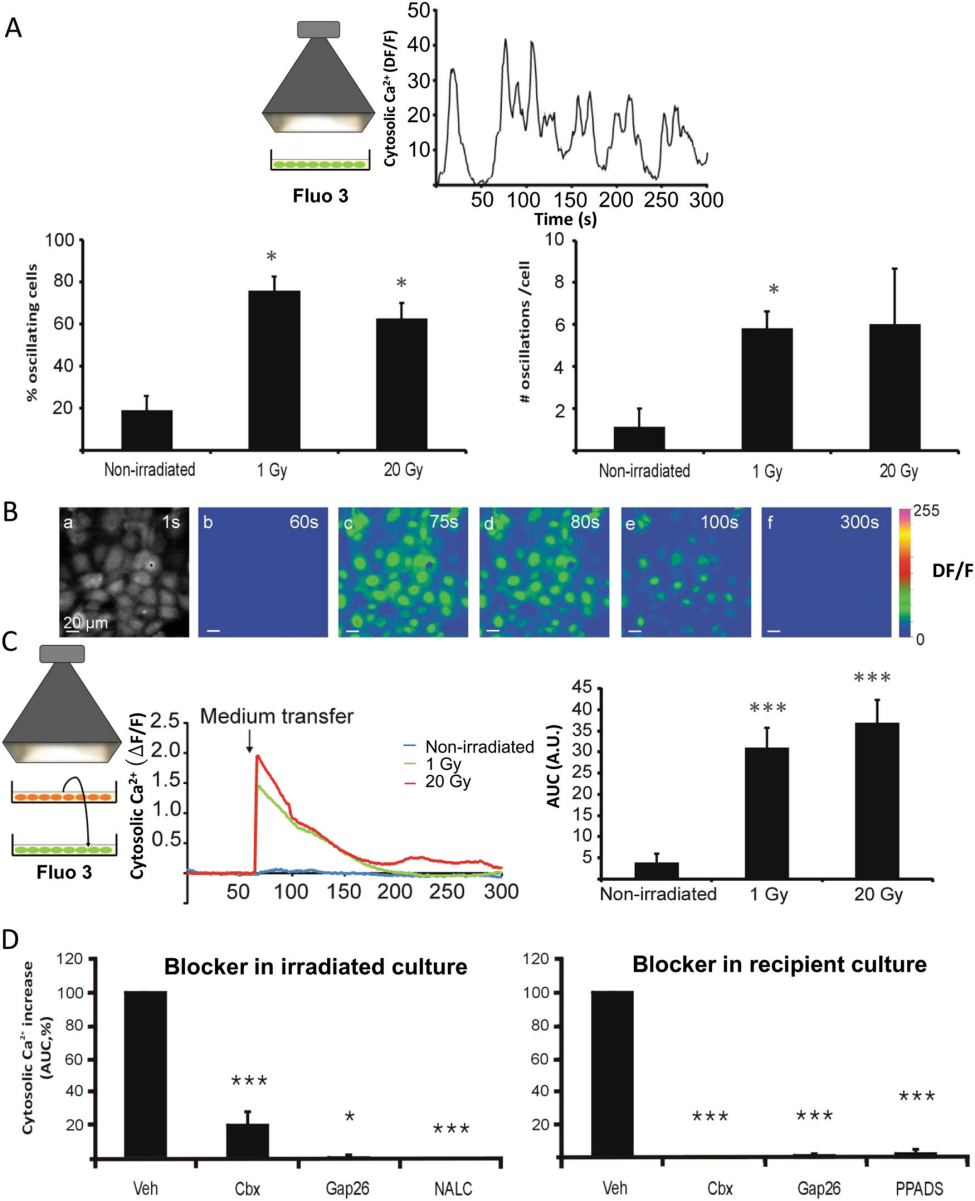
Irradiation triggers cytosolic Ca^2+^ dynamics by direct and paracrine factors. **a** Broad-beam irradiation of RBE4 cells triggers cytosolic Ca^2+^ oscillations recorded 5 min after 1 Gy irradiation. Both the percentage of oscillating cells and the number of oscillations per cell increased upon irradiation. An asterisk (*) vs. non-irradiated (*n* = 2 cell dishes). **b** Cytosolic Ca^2+^ imaging demonstrating Ca^2+^ dynamics in response to medium transfer from 1 Gy broad-beam irradiated RBE4 cells to reporter RBE4 cells loaded with fluo-3-AM (scale bar measures 20 µm). Image **a** shows resting fluo-3 fluorescence; **b**–**f** are Δ*F*/*F* images with **b** just before medium transfer and subsequent images at time points indicated. **c** Broad-beam irradiation and medium transfer as used for the experiment in **b**. Graph shows representative time courses of cytosolic Ca^2+^ changes upon medium transfer. Bar chart right summarizes average values of area under the curve (AUC) of the Ca^2+^ changes, demonstrating irradiation significantly increased the AUC compared with medium transferred from non-irradiated cells. An asterisk (*) vs. non-irradiated (*n* = 3–7). **d** Connexin channel inhibitors added either to the irradiated (left) or recipient cells (right) strongly reduced the Ca^2+^ response in the recipient cells (AUC, expressed relative to vehicle). An asterisk (*) vs. vehicle (*n* = 3–8).

Ionizing radiation has been shown to rapidly generate ROS as well as a rise of cytoplasmic Ca^2+^ (Ca^2+^-signal)^38^, which are two important triggers leading to hemichannel opening^14,35^. We performed live cell Ca^2+^-signal imaging experiments starting 5 min after broad-beam irradiation of RBE4 cells, which demonstrated significantly increased Ca^2+^ oscillations in the cells, both in the percentage of oscillating cells and in the number of oscillations per cell compared with non-irradiated controls; no differences were observed between 1 and 20 Gy (Fig. 4a). In a second approach, transfer of medium collected 5 min after RBE4 irradiation to non-irradiated RBE4 cells induced transient Ca^2+^-signal elevation in the recipient cells (Fig. 4b, c). Addition of Cbx, Gap26, or the ROS scavenger NALC (1 mM) to the irradiated cells (30 min before irradiation) reduced the AUC of the Ca^2+^ transients in recipient bystander cells (Fig. 4d). Addition of Cbx, Gap26, or the purinergic receptor blocker PPADS (50 µM) to the recipient cells, also inhibited the Ca^2+^-signal responses (Fig. 4d).

**Fig. 5:**
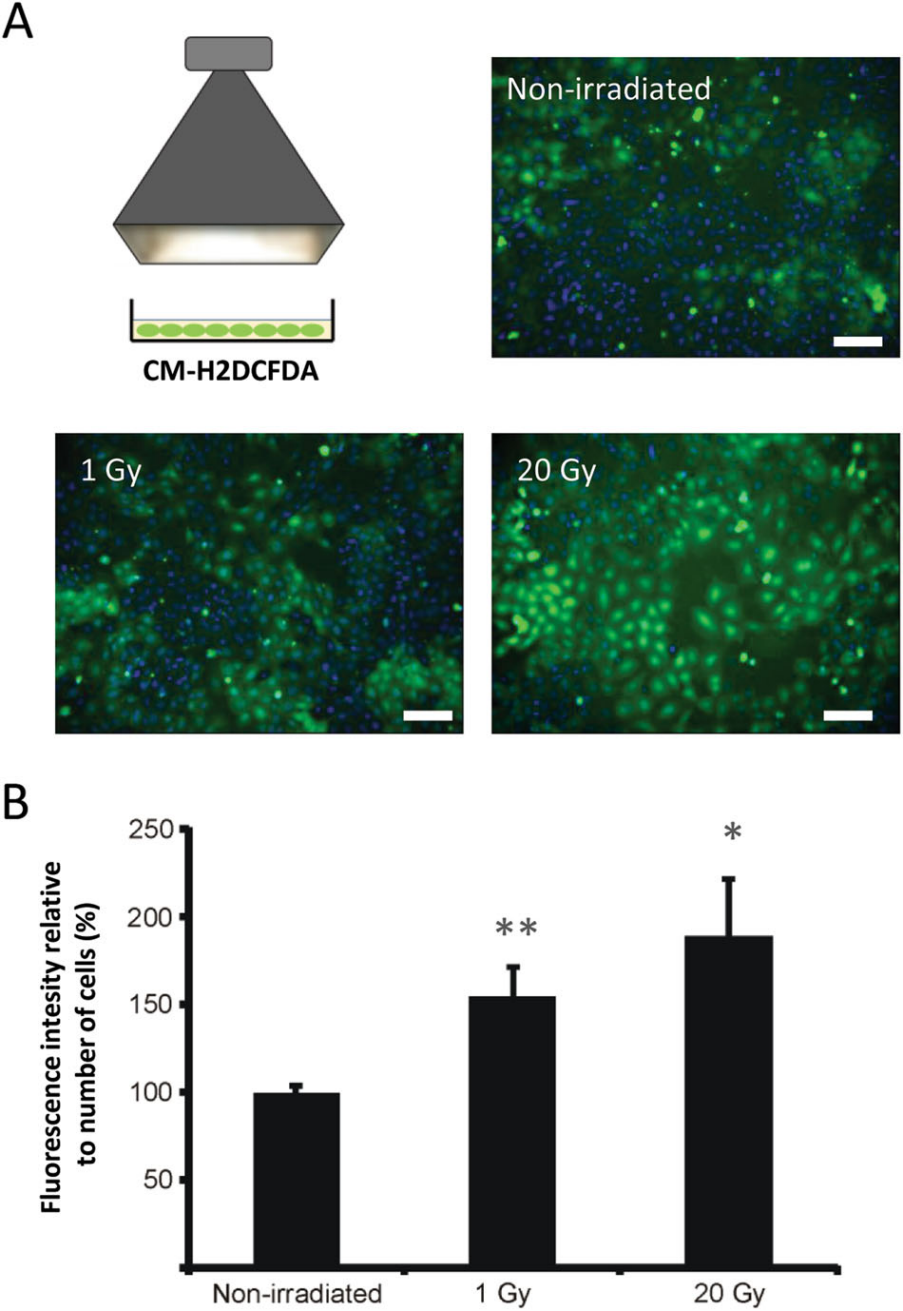
Irradiation triggers ROS production. **a** Broad-beam irradiation increases the fluorescence of CM-H2DFDA-loaded RBE4 cells measured 5 min after irradiation (1 and 20 Gy; scale bar 100 µm). **b** Quantification of fluorescence intensity relative to the nuclei count and normalized to the non-irradiated condition, showing significantly increased signal for 1 and 20 Gy irradiation. An asterisk (*) vs. non-irradiated (*n* = 10–11).

In a third instance, RBE4 cells were preloaded with the oxidative stress marker CM-H2DCFDA-AM, after which the whole cell dish was exposed to X-rays at 1 or 20 Gy. Irradiation induced a significant increase in signal intensity of the CM-H2DCFDA probe measured 5 min after irradiation (Fig. 5). The signal had a trend to increase with dose but this did not attain statistical significance, as observed for the Ca^2+^-signal responses to medium transfer from irradiated cells (Fig. 4c).

**Fig. 6:**
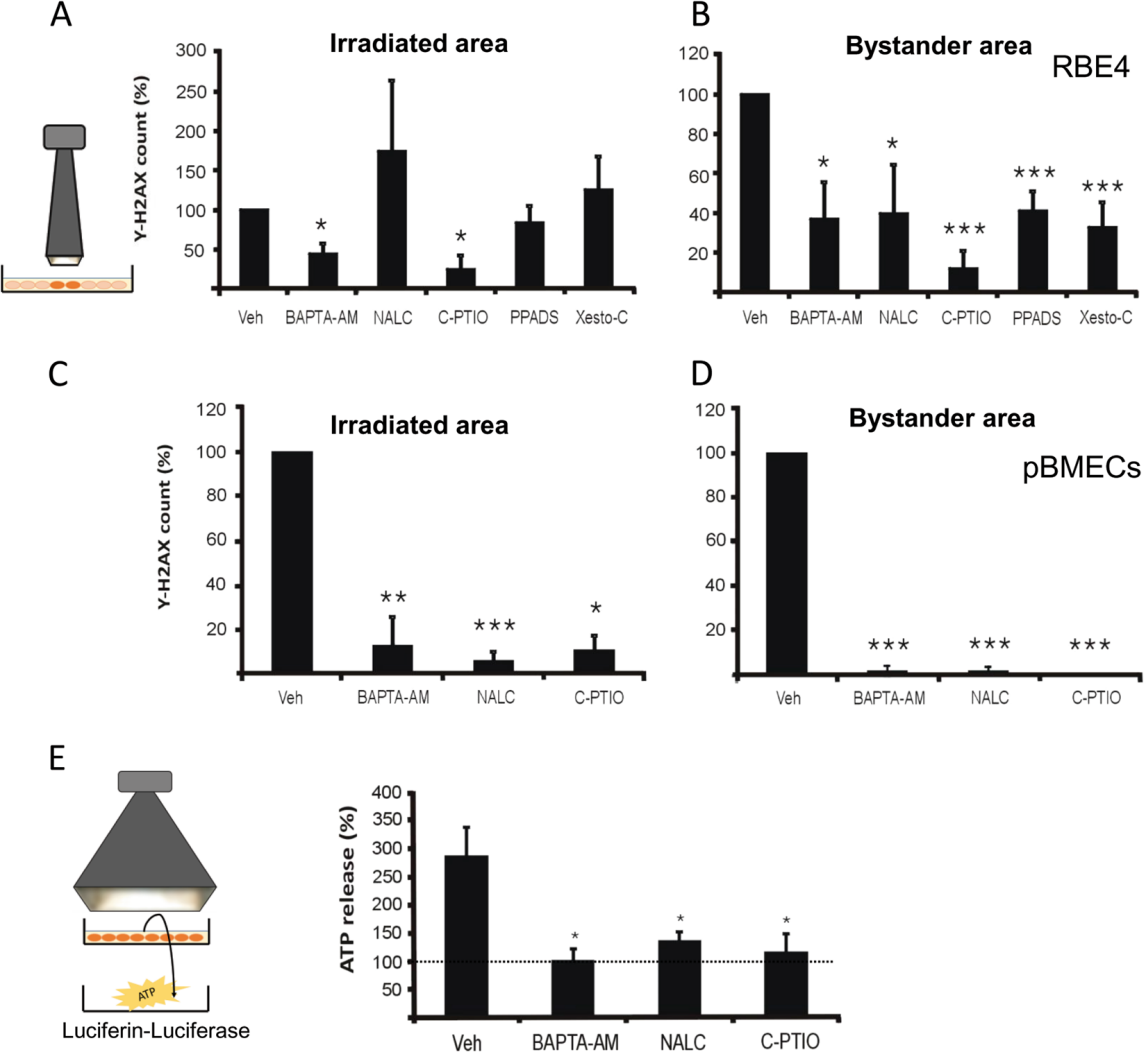
Inhibitors of signaling via Ca^2+^, ROS, NO, ATP, and IP_3_ inhibit γ-H2AX responses in the irradiated and bystander areas. **a** Focused 3 × 3 mm beam irradiation experiments and the effect of cytosolic Ca^2+^-chelation with BAPTA-AM, ROS scavenging with NALC, NO scavenging with C-PTIO, purinergic P2X antagonism with PPADS, and IP_3_ receptor antagonism with xestospongin C (Xesto C) on γ-H2AX counts (normalized to vehicle) in RBE4 cells in the irradiated zone. An asterisk (*) vs. vehicle (*n* = 7–10). **b** Effects in the bystander area. An asterisk (*) vs. vehicle (*n* = 7–10). **c**, **d** Effect in irradiated and bystander areas in pBMECs. An asterisk (*) vs. vehicle (*n* = 4–7). **e** BAPTA-AM, NALC, and C-PTIO also inhibited irradiation-induced ATP release (normalized to non-irradiated control). An asterisk (*) vs. vehicle (*n* = 3–6).

Finally, a contribution of Ca^2+^-signal elevation and ROS production on hemichannel opening was investigated by recording the effect of BAPTA-AM (10 µM; 1 h preloading) and NALC (1 mM; 30 min preloading) on radiation-induced ATP release. Both compounds significantly decreased ATP release 5 min after 1 Gy irradiation (Fig. 6e). NO is involved in the response to ionizing radiation^33,39^ as well as in hemichannel opening^40,41^ and we further tested the effect of NO scavenging by preloading the cells with C-PTIO (100 µM; 30 min prior to 1 Gy irradiation); this strongly reduced irradiation-induced ATP release (Fig. 6e) while having no effect on gap junctional coupling (Supplementary Fig. 3f). Taken together, these findings indicate that the opening of hemichannels is an early step in the response of cells to X-rays which involves Ca^2+^, ROS, and NO signaling pathways.

### Interfering with Ca^2+^, ROS, NO, or ATP signaling reduces irradiation-induced γ-H2AX scores in irradiated and bystander zones

To further document the role of Ca^2+^, ROS, and NO signaling in the generation of γ-H2AX bystander effects, we applied BAPTA-AM, NALC, and C-PTIO before focused irradiation (3 × 3 collimator) and analyzed their effect in the irradiated and bystander areas. These inhibitors significantly reduced the γ-H2AX scores in both areas, most clearly in primary pBMEC (Fig. 6a–d) with less clear responses being observed in RBE4 cells, especially in the irradiated area (Fig. 6a, c). Interestingly, the IP_3_ receptor antagonist Xesto C (5 µM) significantly reduced γ-H2AX counts in the bystander zone of RBE4 cells but not in the irradiated area, suggesting IP_3_ generation in the irradiated zone that is transferred to the bystander zone for subsequent effects. In line with the irradiation-induced ATP release (Fig. 6e), we found that blocking purinergic receptor signaling with PPADS significantly reduced γ-H2AX scores in the bystander area in RBE4 cells (Fig. 6b).

**Fig. 7:**
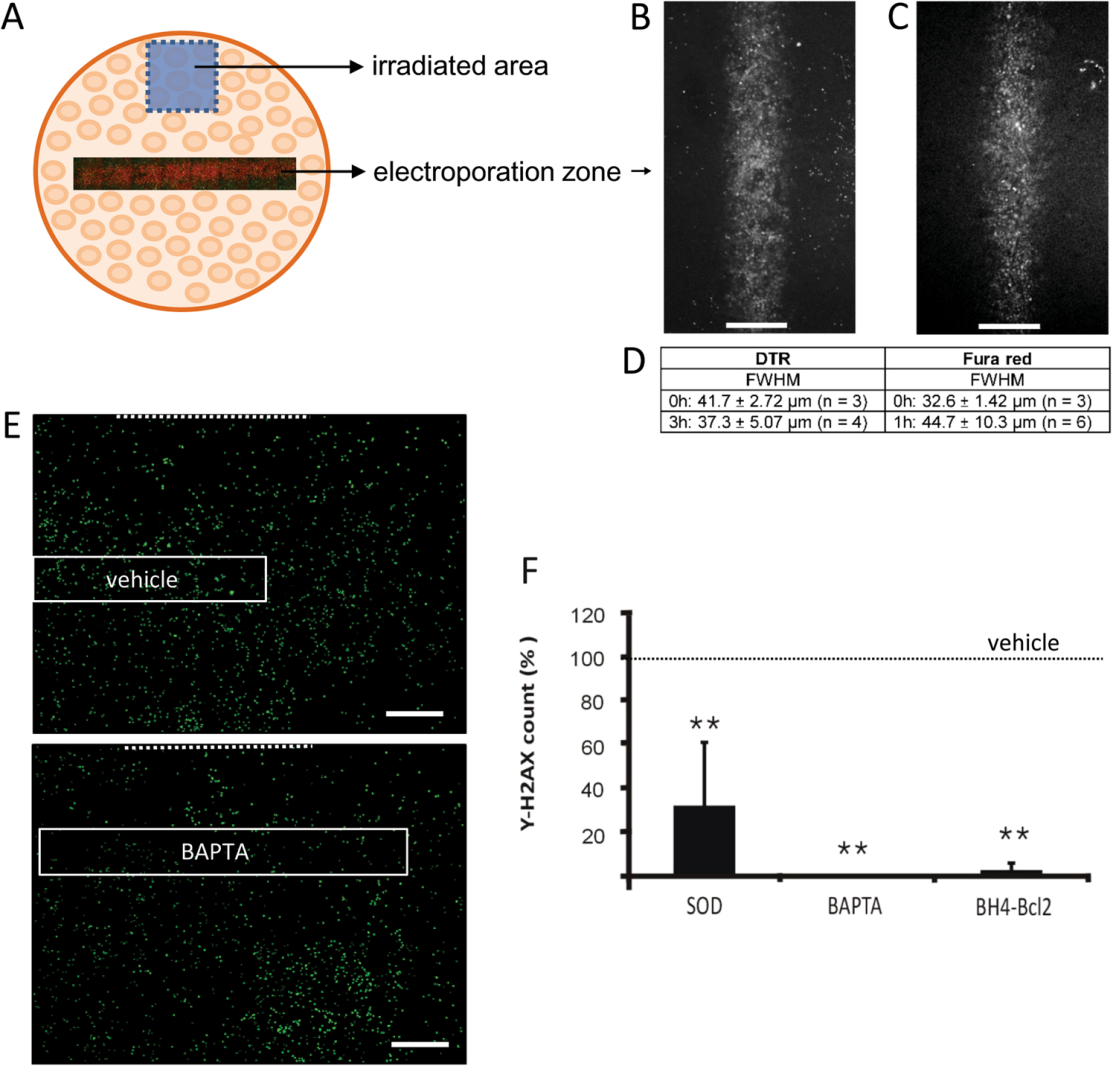
Localized electroporation loading of cells within the bystander zone with cell-impermeable inhibitors of Ca^2+^ and ROS signaling pathways strongly reduce γ-H2AX scores. **a** Schematic overview of the electroporation experiment, with indication of the irradiated zone and electroporation zone located in the bystander area. **b** Fluorescence image of the electroporation zone in RBE4 cells as visualized by 10 kDa dextran Texas red (DTR) immediately after electroporation (0 h time point; size bar 50 µm). **c** Experiment as in **b** but loaded with fura red as a diffusion reporter (0 h time point). **d** Full width at half maximum (FWHM) for both dyes at 0 h and later. **e** Representative γ-H2AX images with indication of the electroporation zone loaded with vehicle or BAPTA (size bar 1 mm). Dotted line indicates the border of the irradiated area. **f** Summary γ-H2AX data in the electroporation zone, demonstrating that SOD, BAPTA, and BH4-Bcl2 significantly decreased γ-H2AX counts compared with vehicle. An asterisk (*) vs. vehicle (*n* = 4–5).

In addition to effects in the bystander area, some of the pharmacological blockers also had significant effects in the directly irradiated area (Fig. 6a, c). Consequently, the reduction of the bystander effects may partly be caused by inhibitory effects in both irradiated and bystander zones. We thus set out to determine the effect of interfering with IP_3_, Ca^2+^, and ROS signaling in the bystander zone only, without exposing the irradiated zone to the inhibitors used. To that purpose, we applied in situ electroporation to load a small and defined zone of cells within the bystander area with membrane-impermeable inhibitors of IP_3_, Ca^2+^, or ROS signaling^42^ (Fig. 7). To interfere with IP_3_ signaling, we loaded the cells with the IP_3_ receptor blocking peptide BH4-Bcl2 (amino acids 6-30 of Bcl2; MW 3.6 kDa; 20 µM), which interacts with the regulatory/coupling domain of the IP_3_R^43–45^. To interfere with downstream Ca^2+^ signaling, we loaded cell-impermeable BAPTA (MW 476 Da in the absence of Ca^2+^) into the cells. For ROS, we used the high MW scavenger SOD (MW 32.5 kDa; 3 µM). Of all these substances, BAPTA is the only substance that may potentially pass through gap junctions and thus spread beyond the loaded cell zone. To visualize the spatial distribution of BAPTA and the other high MW substances, we included fura red (809 Da) and 10 kDa DTR in the electroporation solutions. Immediately after electroporation, the full width at half maximum (FWHM) of the electroporation zone was ~42 µm for the 10 kDa DTR and ~33 µm for fura red (Fig. 7d). For DTR, the electroporated zone was not significantly wider 3 h later (~37 µm). For fura red, the fluorescence was too low to estimate the zone width at 3 h; at 1 h we obtained reliable images giving a FWHM of ~45 µm, which is slightly but not significantly larger compared with the width recorded immediately after electroporation. Based on these results, we reasonably concluded that BAPTA diffusion via gap junctions must be limited. We next quantified the γ-H2AX counts in the zone indicated by the 10 kDa DTR tracer, which was included in the electroporation vehicle solution for all experiments. γ-H2AX scoring was done as in all previous experiments, but here we normalized to the score measured in vehicle-electroporated experiments performed in parallel. Fig. 7f summarizes average data of these experiments, demonstrating that BH4-Bcl2, BAPTA, and SOD all strongly reduced the γ-H2AX scores in the electroporation-loaded bystander zone. The strongest effects were observed for the IP_3_/Ca^2+^ signaling axis (BH4-Bcl2 and BAPTA). In a last step, we investigated the role of IP_3_ diffusion via gap junctions, making use of C6 cells stably expressing the V84L mutant Cx26 that is characterized by strongly reduced gap junctional IP_3_ permeability^43^. Focused collimator-based irradiation of C6-Cx26 V84L mutant cells demonstrated significantly decreased γ-H2AX scores in the irradiated and bystander zones compared with C6-Cx26 WT cells (Supplementary Fig. 3d), indicating that gap junctional IP_3_ diffusion is involved in propagating DNA damage.

## Discussion

The results demonstrate that bystander communication of DNA damage involves connexin signaling via gap junctions and hemichannels, the canonical IP_3_/Ca^2+^ signaling cascade, extracellular ATP, and ROS/NO signaling. Each of these signals has been implicated in radiation-induced bystander signaling^38,39,46–50^, but a coherent framework is currently lacking. Of note, most of these signals play a role in cell–cell communication of Ca^2+^ signals^51^ and all of them have been linked to hemichannels, either as a trigger for their opening^14,40,52,53^ or as a substance released in a manner facilitated by hemichannels^54,55^. Specifically, Ca^2+^ signal communication between cells is mediated by gap junctions that pass IP_3_ and Ca^2+^^56^ and by paracrine signaling that involves extracellular ATP as well as NO released via various mechanisms that include hemichannel-related pathways^51,54,57,58^. Below we discuss the present findings in the perspective of this Ca^2+^ signal communication framework.

Irradiation had dose-dependent effects on γ-H2AX scoring in the directly irradiated zone, while a saturation effect was observed in the bystander area (Fig. 1i), as reported by others^31–33^. Saturation effects in the bystander zone were also observed in the Ca^2+^-signal responses of recipient cells to medium transfer collected from irradiated cells (Fig. 4a), as observed with γ-rays^46,59,60^; superoxide production triggered by α-particles likewise displayed saturation effects^32^. Saturation may result from rate-limiting processes in the signaling cascade and/or regenerative mechanisms that generate maximal signal regardless of the input magnitude. Accordingly, gap junctions may function as a rate-limiting step while regeneration may occur by Ca^2+^ activation of Phospholipase C (PLC) thereby regenerating IP_3_, and by ATP-induced ATP release^51^.

IP_3_ diffusion through gap junctions contributed to bystander signaling as demonstrated by the reduced γ-H2AX spread in C6 glioma cells expressing IP_3_-impermeable Cx26 channels. Cx26 is not expressed in microvascular brain endothelial cells but inhibition of IP_3_ receptors with Xestospongin C (Fig. 6a, b) or BH4-Bcl2 (Fig. 7f) significantly reduced γ-H2AX spreading, pointing to a role for IP_3_ either diffusing through gap junctions or regenerated as a result of paracrine messengers that activate PLC in distant cells. The gap junctional IP_3_ diffusion route is certainly involved as junctional coupling was intact and maintained during the time frame of bystander spreading (Supplementary Fig. 3c, d). Also hemichannels contributed and we found that Cx43 hemichannels were open 5 min post irradiation (Fig. 3). Connexin-linked ATP release triggered by γ-rays has been reported in various cell types^21,48,49^ based on work with non-specific blockers like lindane^48^ or more specific connexin-targeting peptides like Gap26^21^. In vivo evidence from Cx43 heterozygous mice (Cx43^+/−^) exposed to X-rays has furthermore demonstrated a strong linkage between connexin-based ATP release and γ-H2AX bystander responses^61^. Here, we report that Cbx, Gap26, and TAT-Gap19 suppress γ-H2AX appearance in the bystander zone, with stronger effect size and statistical significance as compared with the irradiated zone (Fig. 2b–e). The smaller effect size in the directly irradiated zone is likely the consequence of the fact that cells are directly hit by radiation in this zone. As Cbx also inhibits Panx1 channels (IC_50_ in the range of 2–5 µM; presently used concentration 50 µM) and RBE4 cells express Panx1^62^, we cannot exclude Panx1 channel involvement in the results obtained with Cbx. By contrast, TAT-Gap19, which targets Cx43 hemichannels, has no effect on Panx1 channels^25^ and results obtained with this peptide inhibitor therefore genuinely reflect Cx involvement. Conditional endothelial Cx43 knockout had non-significant effects on bystander communication (Fig. 2f), which may relate to the concomitant increase of Cx37 that can in part compensate for the lost Cx43 (Supplementary Fig. 3b). The strong potency of TAT-Gap19 to inhibit bystander area DNA damage (Fig. 2c, e) indicates a dominant effect of Cx43 hemichannels while general Cx channel block with Cbx proved less effective and of similar magnitude as in endothelial Cx43 knockout (Fig. 2f).

The irradiated cells showed clear oscillatory Ca^2+^-signal dynamics that play a role in hemichannel opening and ATP release^53^. Blocking connexin channels furthermore strongly inhibited Ca^2+^-signal dynamics induced by medium transfer from irradiated cells to recipient naive cells, irrespective whether the inhibitors were applied to irradiated or recipient cell dishes (Fig. 4d). This suggests that connexins, as hemichannels, facilitate the paracrine release of agents that activate Ca^2+^-signal changes in recipient cells^16,63^ and facilitate Ca^2+^ entry at the recipient side^53^. ROS-induced Ca^2+^ entry has indeed been demonstrated to be a crucial factor in bystander effects^59^. In addition to ATP, the irradiated cells also produced ROS (Fig. 5a, b), which may act as a trigger for ATP release^48^; accordingly, ROS inhibition with NALC suppressed ATP release (Fig. 6e). Moreover, NALC inhibition in irradiated cell dishes and PPADS purinergic receptor inhibition in recipient dishes suppressed Ca^2+^-signal changes in recipient cells after medium transfer (Fig. 4d). ROS can influence Ca^2+^ signal by inducing ER Ca^2+^ release, inhibition of plasma membrane/ER Ca^2+^ ATPases, stimulation of Ca^2+^-induced Ca^2+^ release, or mitochondrial permeability transition pore opening^64,65^. Conversely, Ca^2+^ influences ROS signaling in opposite ways: it induces secondary ROS generation in mitochondria via increased oxidative phosphorylation or mitochondrial permeability transition pore opening^46,59,66^, but can also mitigate ROS signaling by activating antioxidant enzymes such as catalase and SOD^64,65^. Taken together, these data indicate a tightly connected ATP-ROS-Ca^2+^-signal signaling triad with strong impact on bystander γ-H2AX responses as judged from the suppressive effect size of PPADS, NALC, BAPTA-AM Ca^2+^-signal buffering and Xesto C inhibition of IP_3_ receptors (Fig. 7a, b). NO may be part of this triad, as it is activated by Ca^2+^-calmodulin signaling, facilitates intercellular Ca^2+^ signal communication^57^, contributes to bystander Ca^2+^-signal signaling in photodynamic therapy^67^, and may induce DSBs via peroxynitrite (ONOO^−^) formed by its reaction with ROS^39^. Accordingly, C-PTIO-based NO scavenging inhibited radiation-induced ATP release (Fig. 6e) as well as bystander γ-H2AX generation (Fig. 6b). Interestingly, the effects of C-PTIO were, like BAPTA-AM and NALC, stronger in pBMECs compared with RBE4, bringing down the γ-H2AX counts to almost zero (Fig. 6a–d); equally strong effects were observed for Gap26 (Fig. 2b–e)^68–71^.

**Fig. 8:**
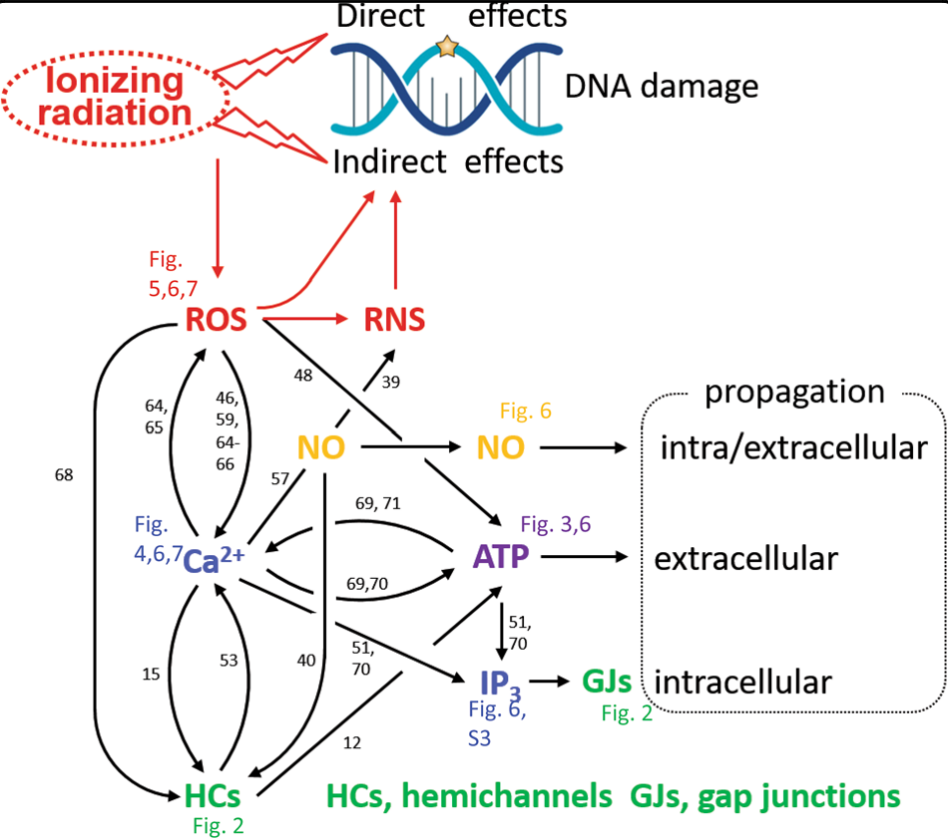
Schematic view of the bystander signal communication network. Ionizing radiation directly interacts with DNA molecules and indirectly via radiolysis of water generating ROS and reactive nitrogen species (RNS) produced by ROS interaction with nitric oxide (NO), leading to indirect DNA damage. ROS elevates intracellular Ca^2+^, and Ca^2+^ on its turn triggers ROS; Ca^2+^ also activates synthesis of NO and IP_3_, and the release of ATP, which drive intracellular and extracellular bystander propagation. IP_3_ and Ca^2+^ pass through gap junctions (GJs) while ATP is released via various mechanisms including hemichannels (HCs) that are opened by ROS, Ca^2+^, and NO. Numbers refer to reference list; Figure numbers refer to results reported here.

All things considered, we propose that irradiation activates ATP-ROS-Ca^2+^-signal signaling with ROS as the primary generated signal, which, given its very short lifetime (10^−9^ s for the hydroxyl radical^23^) and diffusion distance (4 nm for the hydroxyl radical^24^)^72^, has a limited role in long-range bystander consequences. Ca^2+^ hereby acts as an intracellular and ATP as an extracellular propagator of bystander effects. Both ATP and Ca^2+^ diffuse and are actively regenerated by Ca^2+^-activated IP_3_ regeneration^51^ and ATP-induced ATP release^73,74^. Moreover, Ca^2+^ may also contribute as an extracellular signal that enters the cells via membrane channels, including open hemichannels and Ca^2+^ channels. As concerns the role of NO, this messenger has an estimated diffusion distance in the order of 160 µm^24^ and may be involved in propagation but this needs to be balanced with the fact that low NO concentrations may also mitigate bystander effects^75,76^. During propagation, extensive crosstalk between these messengers will effectively assemble a robust signaling network linked by hemichannels or gap junctions (Fig. 8). Collectively, the data indicate feed-forward propagation of secondary ROS generation in the bystander zone, driven by the IP_3_/Ca^2+^ signaling axis and leading to DNA damage, as apparent from the local interference with ROS/IP_3_/Ca^2+^ signaling in the bystander zone (Fig. 7f). Mitochondria likely contribute to secondary ROS generation, as documented for bystander communication of DNA mutations, autophagy, and apoptosis^77^, which may involve Ca^2+^-dependent mitochondrial permeability transition^78^. As targeting IP_3_/Ca^2+^ signaling in the bystander zone tended to be more efficient in preventing DNA damage than targeting ROS (Fig. 7f), direct Ca^2+^/calmodulin-dependent effects on chromatin structure^79^ may additionally contribute.

Significance for the field. Brain irradiation after glioblastoma tumor removal not only targets remaining tumor cell niches but also brain vascular endothelial cells that, given their close presence to blood oxygen, are highly susceptible to radiation damage. As a result, bystander effects propagating along the network of microvascular endothelial cells and negatively affecting their function or leading to various modes of cell death, will disturb vascular function, resulting in ischemic alterations that compromise normal brain function in the involved areas. Brain endothelial cells not only form the capillary blood–brain barrier but also interact with numerous other cell types, including blood cells, vascular wall cells (pericytes, smooth muscle cells), and parenchymal cells (microglial cells, astrocytes and neuronal cells)^80^ the interaction with which may be altered at bystander distance, resulting in disturbed neuro-glio-vascular unit functioning.

## Supplementary information

supplementary material
