# Supplementary material for: Cx43 channels and signaling via IP3/Ca2+, ATP, and ROS/NO propagate radiation-induced DNA damage to non-irradiated brain microvascular endothelial cells
Source: Cell Death Dis. 2020 Mar 18;11(3):194. doi: 10.1038/s41419-020-2392-5 (PMC7080808; doi:10.1038/s41419-020-2392-5)
Supplement: Supplementary file 1 — supplementary material [file 41419_2020_2392_MOESM1_ESM.pdf]

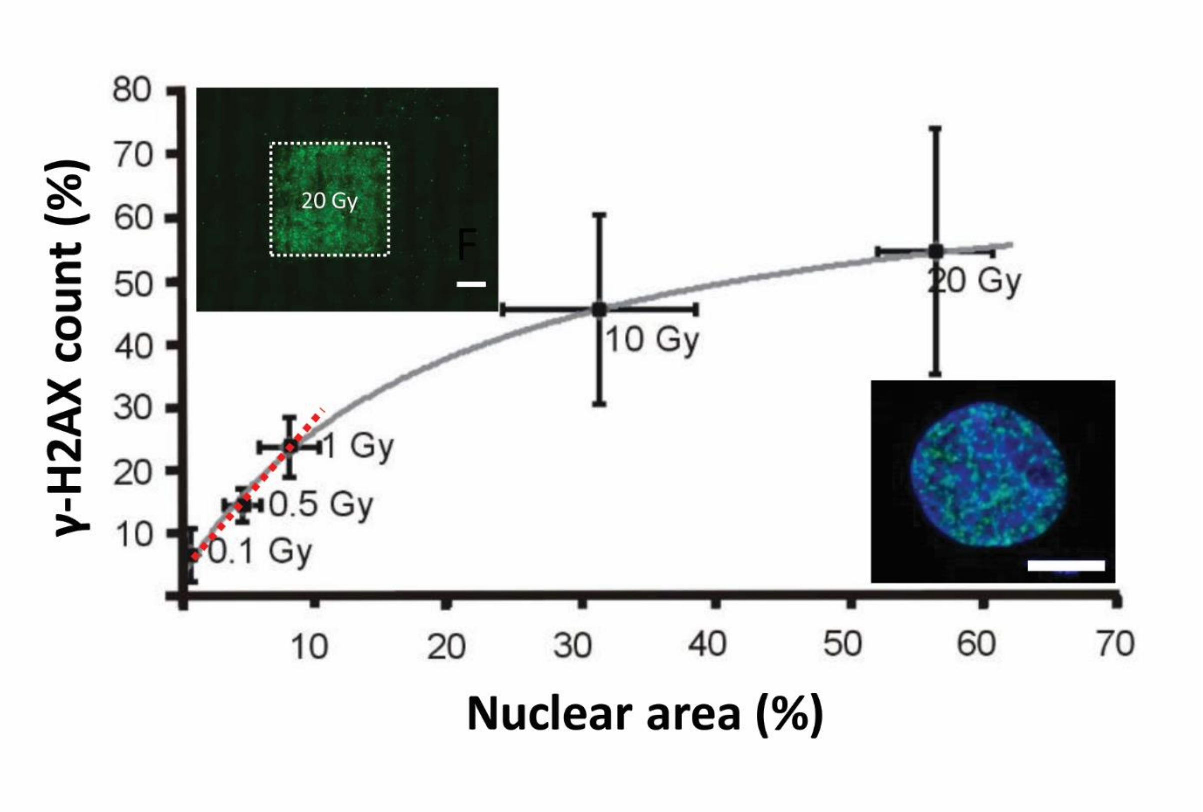

Supplementary Figure 1: Relation between low and high magnification analysis of γ-H2AX foci counts in the irradiated zone.

The y-axis shows γ-H2AX counts in the irradiated area determined from images recorded with a x10 objective (scale bar 1 mm) whereas the x-axis shows the percentage of γ-H2AX stained surface area relative to the nuclear surface area based on images acquired with a x63 objective (scale bar 10 µm) (n = 2-3). The relation is initially linear (red dotted line) and flattens at higher doses, resulting in an underestimation of the γ-H2AX counts in the irradiated zone for the 20 Gy dose.

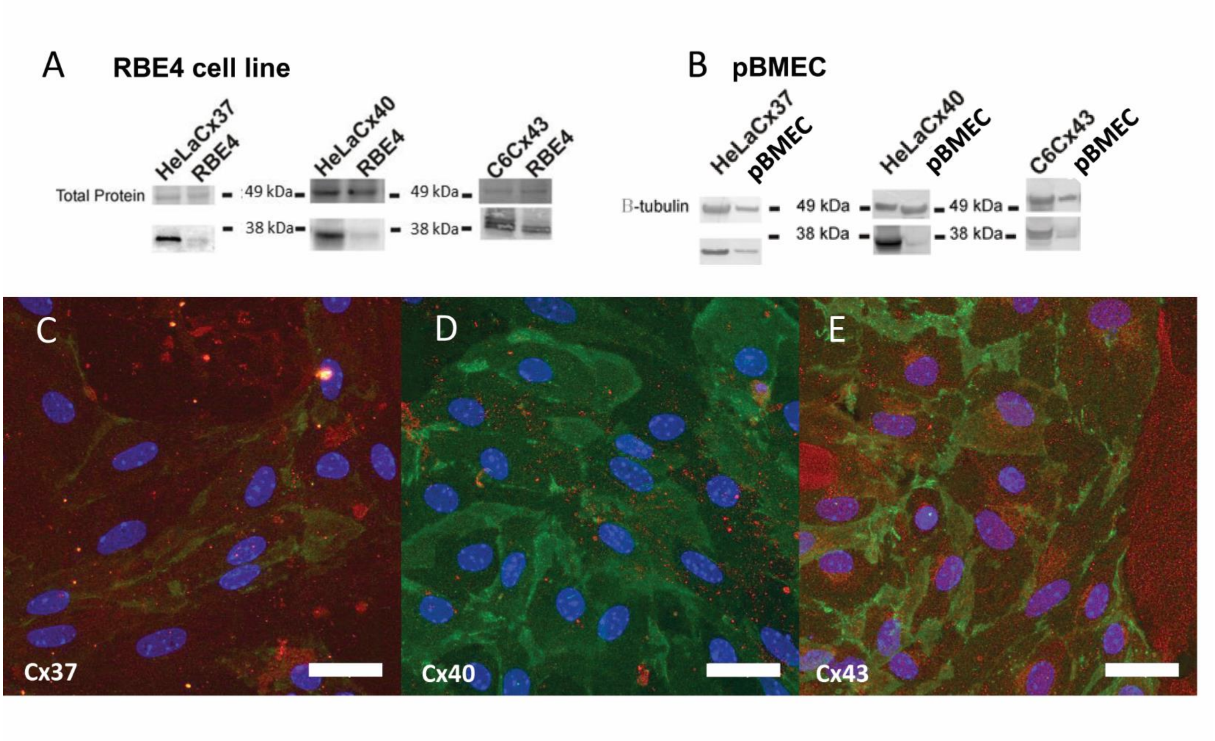

Supplementary Figure 2: Connexin expression in brain microvascular endothelial cells.

A. Western blotting analysis demonstrating Cx43, Cx40 and Cx37 expression in RBE4 cells. Total protein was determined by sypro staining. B. Primary BMECs, also expressed the three Cxs. Beta-tubulin was used as a loading control. C-E. Representative immunostainings demonstrating Cx37 (C), Cx40 (D) and Cx43 (E) in red, CD31 in green and DAPI nuclei in blue (scale bar 80 µm).

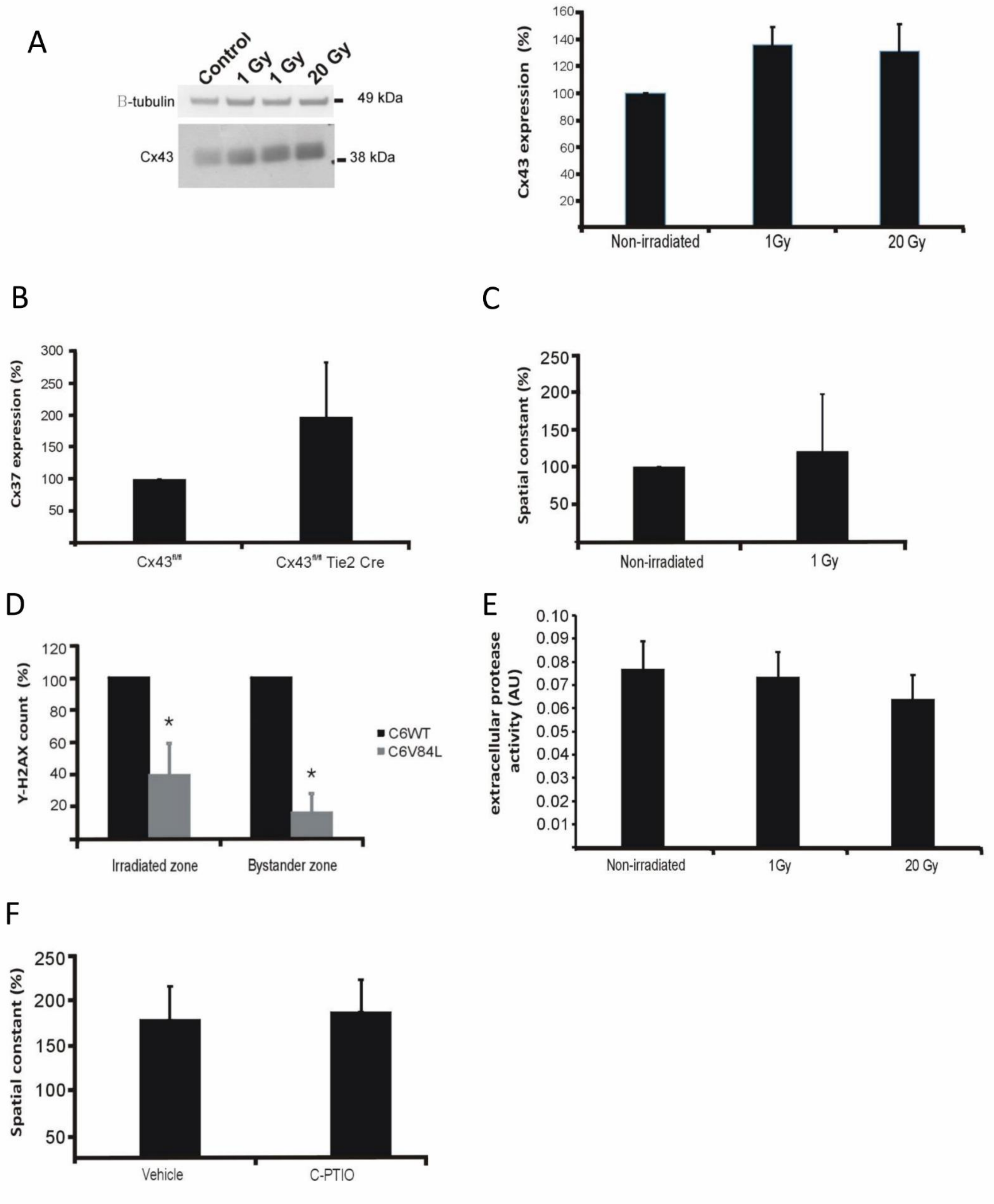

Supplementary Figure 3: Connexin expression, gap junctional coupling and other control experiments

A. Cx43 expression in RBE4 cells shows a trend to increase following irradiation with 1 and 20 Gy. B. Cx37 expression is increased in pBMECs isolated from C57BL/6 Cx43fl/fl:Tie2-Cre+mice compared to Cx43fl/fl control animals (n = 6, not significant). C. Gap junctional coupling studied by scrape loading and dye transfer (SLDT) quantified by the spatial constant of 6-carboxyfluorescein dye spread (normalized to non-irradiated; n = 6). Irradiation with 1 Gy did not significantly alter dye spread. D. Experiments on C6 WT cells and stably expressing mutant Cx26 with reduced IP_3 permeability (C6V84L) (n = 7), demonstrating significantly reduced γ-H2AX counts in the irradiated and bystander areas. E. Extracellular protease activity (AU) in non-irradiated and broad-beam irradiated cells (1 and 20 Gy), 5 min post irradiation (n = 10). F. Gap junctional coupling quantified by the spatial constant of 6-carboxyfluorescein dye spread in cells treated with vehicle or 100 μM C-PTIO (n = 7).
